# Feasibility study of the effects of art as a creative engagement intervention during stroke rehabilitation on improvement of psychosocial outcomes: study protocol for a single blind randomized controlled trial: the ACES study

**DOI:** 10.1186/1745-6215-15-380

**Published:** 2014-09-28

**Authors:** Jacqui H Morris, Chris Kelly, Madalina Toma, Thilo Kroll, Sara Joice, Gillian Mead, Peter Donnan, Brian Williams

**Affiliations:** Social Dimensions of Health Institute, University of Dundee, 11 Airlie Place, Dundee, DD1 4HJ UK; Art as Creative Engagement Intervention for Stroke (ACES) Study Manager, NHS Tayside, Kings Cross, Clepington Rd, Dundee, DD3 8EA UK; School of Psychology, Te Kura Hinegaro Tangata, Massey University, Private Bag 11 222, Palmerston North, Aotearoa New Zealand; Royal Infirmary of Edinburgh, Little France Crescent, University of Edinburgh, Room S1644, Edinburgh, EH16 5NN UK; Population Health Sciences, Mackenzie Building, Kirsty Semple Way, Dundee, DD2 4BF UK; Nursing, Midwifery and Allied Health Professions Research Unit, Scion House, University of Stirling, Stirling, FK9 4NF UK

**Keywords:** Stroke, Art, Mood, Self-esteem, Health status

## Abstract

**Background:**

Benefits of art participation after stroke are becoming increasingly recognized. Qualitative studies suggest that participation in visual arts creative engagement interventions (CEIs) during rehabilitation after stroke may improve mood, self-esteem, hope and some aspects of physical recovery. This study examines the feasibility of undertaking a randomized controlled trial of a CEI delivered by artists within in-patient stroke rehabilitation to test effectiveness.

**Methods/Design:**

This trial is a two arm, single-blind, randomized controlled feasibility trial within in-patient stroke rehabilitation. We will recruit 80 patients receiving stroke rehabilitation in two stroke units in a health board area of Scotland (40 patients in each arm). Intervention arm participants will receive a visual-arts based CEI facilitated by experienced artists. Artists will follow an intervention protocol with specific components that enable participants to set, achieve and review artistic goals. Participants will receive up to eight intervention sessions, four within a group and four one-to-one with the artist. Control group participants will receive usual care only.

Data collection will occur at baseline, post-intervention and three-month follow-up. Stroke-related health status is the primary outcome; mood, self-esteem, self-efficacy, perceived recovery control and hope are secondary outcomes. Semi-structured interviews will be conducted with purposively selected patients, artists and healthcare staff to elicit views and experiences of the intervention and feasibility and acceptability of trial processes. Recruitment rates, retention rates and patient preference for art participation will also be collected. Data will indicate, with confidence intervals, the proportion of patients choosing or refusing participation in the CEI and will allow calculation of recruitment rates for a future definitive trial. Summary data will indicate potential variability, magnitude and direction of difference between groups. Findings will inform sample size calculations for a definitive trial. Thematic analysis of qualitative data will be managed using the Framework Approach. Framework is an analytical approach for qualitative data, commonly used in policy and medical research.

**Discussion:**

If shown to demonstrate effects, this intervention has the potential to address aspects of stroke recovery previously. Not routinely addressed in rehabilitation.

**Trial registration:**

Registered with Clinical Trials.Gov: NCT02085226 on 6th March 2014.

**Electronic supplementary material:**

The online version of this article (doi:10.1186/1745-6215-15-380) contains supplementary material, which is available to authorized users.

## Background

More than 150,000 people in the United Kingdom experience a new stroke per year [1]. Stroke is the main cause of complex adult disability [2], with more than 300,000 people living with its effects at any time. As many as 85% of stroke survivors experience hemiplegia, which leads to impaired motor control on the affected side of the body. In addition, survivors often experience cognitive and communication impairments that, combined with physical impairments, lead them to experience loss of independence in activities of daily living and restricted return to participation in life roles [1].

Consequently, many survivors experience negative psychosocial consequences of the stroke that further compound its effects. Depression and anxiety are common, affecting 28 to 33% of stroke survivors at any time [3]. These mood disorders are associated with lower levels of independence in activities of daily living [4–6]. Additionally, survivors with low self-esteem experience lower functional status and higher levels of depression than those with more positive views of self [7, 8]. Unsurprisingly, many survivors also report low confidence or self-efficacy in everyday life that is also linked to depression, and loss of independence in activities of daily living [9]. The inherent consequences of these psychosocial effects of stroke and their impact on wider recovery mean that quality of life after stroke is significantly reduced [10]. It is therefore imperative to address these psychosocial consequences, both for the patient and family members

Post-stroke in-patient rehabilitation, typically involving professionals such as physiotherapists, occupational therapists and speech and language therapists, aims to restore physical functioning and communication to enable survivors to return home. However, despite advances that have improved survival rates and recovery of physical independence [11], rehabilitation remains a task-orientated process, focusing on practical daily living. Additionally, rehabilitation tends to adopt a deficit reduction model that focuses on what survivors cannot do, rather than on what they can do [12]. The need to address the psychosocial consequences of stroke are, however, being increasingly recognized and several policy documents now view their management as extremely important, emphasizing the need to improve life after stroke [13–15]. The potential role of adjunct rehabilitation interventions to address psychosocial problems and improve post-stroke quality of life should therefore be explored.

There is growing and convincing evidence that engaging in creative arts activities can promote improvements in perceived physical and mental health, social functioning and wellbeing in long-term conditions. Creative arts programs positively contribute to the wellbeing of people with mental health problems [16–19], cancer [20], dementia [21], brain injury [22] and diabetes [23]. Furthermore, the importance of arts in health is reflected in a recent United Kingdom Department of Health report that emphasized the importance of patient engagement in creative arts for the delivery of better health and wellbeing, and for improved healthcare experiences [24].

For stroke survivors, arts programs have the potential within rehabilitation to address some of the psychosocial consequences of stroke and to improve post-stroke quality of life [25]. A number of programs have been implemented to provide opportunities for survivors to participate in the creation of artwork using a range of visual arts media. Professional artists lead the programs [25, 26] and the focus of the activities is on the positive influence of engagement and participation in the art form. Activities are purely creative and do not involve specific assessment and treatment of psychological status normally undertaken by art therapists. The growing interest in visual arts creative engagement interventions (CEI) as adjunct interventions to traditional healthcare is increasingly reflected in stroke literature. Recent qualitative studies have illustrated the perceived positive impact of creative interventions on achievement of rehabilitation goals and outcomes, identity, mood and wellbeing during post-stroke rehabilitation [26, 27].

Visual art interventions may achieve these effects by enhancing control over recovery through development of confidence, leading to enhanced self-esteem [25, 26]. Theoretically, participation in a CEI may enable patients to make sense of their condition, in line with effects of expressive writing in acute and long-term conditions [28, 29]. Improved self-efficacy, perceived control and autonomy over the illness through creative activity are other ways in which the intervention might work to improve psychosocial outcomes after stroke [23].

However, whilst existing qualitative studies illustrate the perceived benefits and value of CEI to stroke survivors [25–27], the effects of the intervention on specific psychosocial outcomes after stroke has not yet been empirically tested. In health service settings that increasingly face financial challenges, empirical evidence from robust randomized controlled trials (RCT) that demonstrate the effectiveness of art on meaningful clinical outcomes is vital if CEI is to be incorporated as a routine adjunct intervention to rehabilitation.

Undertaking an RCT to evaluate the effects of a CEI on psychosocial outcomes is, however, challenging and a number of uncertainties need to be clarified before proceeding to a full-scale phase III trial. Firstly, the feasibility of undertaking a trial of a non-traditional intervention in a rehabilitation setting needs to be established. Art is an intervention in which preference for participation may strongly influence participant recruitment and attrition and therefore, before proceeding to a full trial, it is vital to identify the role of preference in recruitment and trial participation. Secondly, components of the intervention and their mechanisms of action need to be made explicit. Thirdly, the precise outcomes likely to be influenced by the intervention and corresponding appropriate measures that can be completed by stroke survivors need to be identified.

Selection of appropriate outcome measures is a particular challenge to researchers designing studies to measure the intervention effects of CEI. This is challenging given that the exact mechanisms by which art may influence psychosocial outcomes have not been fully established for stroke survivors. The present trial design is derived from an initial qualitative study that aimed to identify for the purposes of the trial, the components, mechanisms of action and perceived effects of an existing visual art CEI for stroke survivors receiving in-patient rehabilitation. Findings from the qualitative phase were used to design the feasibility trial intervention protocol and identify the outcomes to be evaluated to test the effects of the intervention. The purpose of the present study is to test trial processes to identify feasibility issues in running a CEI, to identify the primary outcome measure and to provide an assessment of effect size for power calculations for a full-scale Phase III trial. The purpose of this manuscript is to describe the trial design and protocol for a feasibility trial.

### Study aims

The aim of this study is to examine the feasibility of conducting an RCT to assess the potential effectiveness of a CEI to improve psychosocial health outcomes in patients receiving in-patient rehabilitation after stroke. To inform the development of a future Phase III trial, we will examine rates of recruitment of patients within in-patient rehabilitation wards to the trial. We will also examine the impact of preference for art on recruitment, retention and outcomes, and explore the acceptability and usefulness of the intervention from the perspective of patients and health professionals. A final aim is to estimate completion rates and effect sizes for relevant patient outcomes to inform the selection of a primary outcome measure and sample size calculations for a full-scale trial.

### Specific objectives

The objectives of this study are to examine the feasibility of intervention delivery in a trial context in two stroke rehabilitation units; to examine the feasibility of the trial processes, recruitment, retention and outcome assessment; and to provide estimates of the effect size of the intervention compared to usual care, in order to inform the development of a full-scale trial

## Methods/Design

We will conduct a single-blind randomized controlled feasibility trial [30]. People with a diagnosis of stroke consecutively admitted to two stroke rehabilitation units in northeast Scotland will be screened for trial inclusion within one week of their admission to rehabilitation. Those meeting inclusion criteria and agreeing to participate will be randomized to an intervention group who will receive the CEI for up to eight sessions with an artist, or a control group that will receive an art portfolio to view. Outcomes will be examined at the end of the intervention and at three-month follow-up.

The rehabilitation units provide rehabilitation services to stroke survivors following discharge from acute stroke units. Admission to a rehabilitation unit typically occurs less than two weeks after stroke onset, and the average rehabilitation stay is 35 days. In the rehabilitation unit, survivors receive occupational therapy, physiotherapy and speech and language therapy as part of their usual care. The CEI will be additional to those therapies

### Ethical approval

This clinical trial will be conducted according to NHS research governance requirements. Patients who agree to allow the study team to access their clinical records will provide written informed consent. All patients will be made aware that they can withdraw from the research at any time. The East of Scotland Research Ethics Committee approved the study (reference number: 13/ES/0006). NHS Research and Development approval has been obtained from NHS Tayside (reference number: 2011GM01). NHS Tayside and the University of Dundee have provided a joint sponsorship agreement.

### Eligibility

#### Inclusion criteria

All stroke survivors admitted to rehabilitation will be eligible for inclusion if they are medically stable and referred for rehabilitation - evidenced by ability to participate in usual rehabilitation therapies; able to sit upright in a chair, supported or unsupported; and if the multidisciplinary team estimates that they will remain in the unit to receive three weeks or more rehabilitation.

#### Exclusion criteria

Stroke survivors will be excluded from the study if medical records report a diagnosis of transient ischaemic attack; if they have acute medical need, are unconscious or medically unwell as evidenced by inability to participate in usual rehabilitation activities; or if they are unable to provide informed consent.

### Recruitment

Nursing and rehabilitation staff will identify potentially eligible patients within one week of admission to either of the stroke unit research venues, or when patients are medically stable and able to participate. The nursing and rehabilitation staff will provide and explain information leaflets to patients about the study, with an invitation to participate. One of two study researchers will approach patients who indicate interest in participating. The study will be fully explained, questions will be answered and initial screening for participation based on communication and comprehension will be will be conducted. Written informed consent for participation will be obtained. Ability to provide informed consent will be guided but not limited by scores on the following measures:

#### The Montreal Cognitive Assessment

The Montreal Cognitive Assessment (MOCA) [31] is a 16-item screening assessment for mild cognitive impairment that was developed in response to the poor sensitivity of the Mini-Mental State Examination for detection of mild cognitive assessment. It is widely used in clinical practice and in studies involving stroke survivors and assesses performance attention, concentration, executive functions, memory, language, visual-constructional skills, conceptual thinking, calculations and orientation. It has good reliability in people with mild cognitive impairment, with an internal consistency of alpha = 0.83 and high test-retest reliability (r = 0.92) [31]. In the same study, high correlation with the Mini-Mental State Examination was also demonstrated (r = 0.87), indicating high criterion validity. The maximum score is 30 and scores of under 26 indicate cognitive impairment. We will use scores that are routinely collected by clinical healthcare staff to assess cognitive impairment.

#### The National Institutes of Health Stroke Scale

The National Institutes of Health Stroke Scale (NIHSS) [32] is a 15-item scale assessing neurological outcomes and degree of recovery after stroke. It assesses motor and sensory function, coordination, language, speech and hemi-inattention. It has established reliability and validity for use in clinical research. Its’ test-retest reliability is high, with mean kappa values between 0.66 and 0.77 [33]. The construct validity of the NIHSS has been demonstrated (r = 0.94) [33], and correlations with lesion volume have been demonstrated to be significant r = 0.37 (P < .001) [34]. The maximum score is 42 with higher scores reflecting greater severity of impairment. Scores of more than one on the communication or consciousness scales will be used as potential indicators of ability to provide informed consent. As well as communication, the scale provides a general assessment of stroke severity and will be used as a baseline characteristic.

### Randomization

Randomization to a control group or an intervention group will be conducted using a remote web-based concealed computer-generated randomization system organized through Tayside Clinical Trials Unit (TCTU). To minimize the likelihood of imbalance between groups, for each site we will stratify according to age (≤60 years, 61 to 80 years, and ≥81 years), gender and disability. Disability will be assessed using routinely collected data from the Barthel Index [35], a scale measuring independence in activities of daily living. The maximum score is 100, indicating independence. Participants will be stratified according to scores that indicate likelihood of independence in activities of daily living (0 to 40, 45 to 55, 60 to 100) [36]. Tayside Clinical Trials Unit (TCTU) will set up a password-accessed online randomization system that will be accessed only by research staff. After baseline assessment, the study researcher will enter stratification details. The system will determine random allocation after which the artists will be informed.

### Intervention

The CEI protocol for the trial has been developed from an existing art program. Tayside Healthcare Arts Trust has delivered a creative engagement program involving visual art to stroke survivors receiving in-patient rehabilitation in Tayside over several years - the Tayside Creative Engagement Intervention (TCEI). The intervention is delivered by artists who are experienced in working with patient populations to facilitate creation of artwork. To prevent contamination with the current trial, that program, which provides one 12-week intervention annually in each unit, will be suspended for the trial duration.

We used data from qualitative interviews with stroke survivors, artists and rehabilitation staff who had previously participated in TCEI to define the intervention components and to model the TCEI for use in the trial. The qualitative phase enabled us to define five discrete intervention stages and their components, and to identify the outcomes likely to be associated with each stage. The stages provide a framework within which the artist’s work, whilst still allowing them to adapt the specific art activities and materials to the needs and interests of individual participants.

Stage 1 involves meeting with artist to discuss interests, stroke and define initial creative goals. Participants’ interests and preferences will be discussed, and knowledge about their state of health and stroke-related impairments will be obtained and recorded in a participant profile. In Stage 2, participants will be introduced to materials and mark making (drawing, collage, printing, painting and mixed-media techniques). This stage will ascertain participants’ ability to handle art materials and their expectations of the process, and provide them with an introductory experience of working with materials. Stage 3 enables participants to move from materials and mark making to developing personal project ideas and goals. Here the artist will guide the participant in use of the materials and how to consider content or subjects that are of personal interest to them. In Stage 4, the participant will control and direct the expression of content and the artist will instruct and facilitate the creative process of interpretation to allow development of personal project ideas into creative finished pieces. Finally, in Stage 5 the artist and participant review the completed work; and the artwork is mounted and displayed within the ward setting for viewing by staff, relatives and other patients. The achievement of a completed creative piece of work to provide a tangible output will lead to the discussion of further ideas which can be progressed by repetition of the intervention stages, supported by the artist. The artist protocol is described in Additional file 1.

### Artist training

Two professionally qualified visual artists who have previously delivered participatory arts programs in healthcare settings will provide the intervention. The artists involved in delivering the intervention have previously delivered arts programs in healthcare settings. Prior to the commencement of the study, the artists will be introduced to trial procedures and the structured intervention in a workshop setting. The artists will be trained in each stage of the intervention protocol and in how goals are developed, plans for achievement made and progress reviewed during each intervention stage. The artists will be required to record participants’ attendance, intervention stage and progression, and record observations of all processes and material and equipment use. The artists will also be trained in the completion of study documentation and the need to maintain blinding. The research manager, a study co-applicant and artist with 15 years of experience working in healthcare settings with stroke survivors and other clinical populations will deliver their training along with the study researchers.

### Intervention procedures

Stage 1 of the intervention involves a discussion and exploration of participants’ interests, preferences, and physical impairments, which the artist will record in a participant profile. This background information will be used to negotiate initial creative goals. The artist will use their expertise and training to collaborate with the participant, to tailor the equipment and media to participants’ impairments and preferences for the topic and nature of the work they wish to undertake. Preferences, based on participants’ interests, occupation, life experiences and previous experience of art will be explored in the initial session and at each subsequent point of progression to ensure that the materials and the topic for creative exploration, is tailored to the individual.

Materials will be individually selected to match participants’ impairments based on their physical ability to handle the materials and their ability to comprehend a process (for example, reverse text in printmaking) to produce a piece of artwork. A range of visual arts materials will be available to the artist and participant, including drawing and painting media, printmaking equipment, three-dimensional modelling clay and craft-based construction materials. These materials present different approaches to art-making that the artist will tailor to match participants’ interests, abilities and impairments. The approaches include two-dimensional mark making using pencils or paintbrushes on paper, two-dimensional printmaking, three-dimensional modelling and three-dimensional craft techniques (weaving, felting and mosaic). All materials and equipment are available from a good online arts and crafts retailer. Modifications to the materials or provision of supplementary equipment will be undertaken as necessary to enable the participants to handle them (for example, providing pencils of brushes of wide diameter to facilitate grip, provision of left-handed scissors or use of anti-slip mats to facilitate one-handed working).

Artistic goals will be set and jointly reviewed by the artist and the participant at each of the intervention stages, and the processes of art-making and the nature of the finished product will be tailored according to progress. To achieve goals, each participant will receive a maximum of four group and four one-to-one sessions with the artist. Group sessions were selected because the social benefits of group interaction during art have previously been shown to be effective [25, 27]. There is also evidence that one-to one sessions with the artist are beneficial for engagement [26]. For these reasons, both modes of delivery will be selected. Participants will engage with the artist over three to five weeks to a maximum of eight sessions. This duration was selected as it reflects the average length of stay of stroke patients in these rehabilitation centers. Participants will have the opportunity to attend at least two sessions per week. The individual sessions will last up to one hour and the group session will last up to one hour and 30 minutes.

During these sessions participants will be progressively taken through the five intervention stages, within which activities and art materials will be adapted and artwork progressed according to personal interests and abilities. Participants may repeat one or more stages a number of times during the course of the intervention duration. When the participant achieves stage 5 in the intervention the artist will review that work and negotiate with the participant to agree at which of the five stages the participant would like to next engage. This may be stage 2 (exploring different materials), stage 3 (developing a new idea or theme) or stage 4 (further work based around an already established idea or theme).

The artists will monitor participants’ progress closely throughout the trial and complete a sessional log of progress, events, material use and other pertinent observations. This log will be reviewed on a monthly basis to establish if any intervention modifications are required. The completion of study documentation will also be monitored on an ongoing basis by the research manager and research associate and will be assessed and reported at the end of the study.

The intervention will take place within two inpatient stroke rehabilitation units at a large wheelchair accessible table(s) in an appropriate location not used for other rehabilitation activity or therapy. All equipment utilized will also be appropriate to this setting (table-top easel, cutting mat and so on). Sessions will be delivered face-to-face by the trained artist as one-to-one sessions with individual participants, between Monday and Friday. To ensure that art participation does not encroach on individual rehabilitation therapies (physiotherapy, occupational therapy and speech and language therapy), which are delivered Monday to Friday, the group sessions with up to a maximum of five participants, will be convened at the weekend, depending on recruitment rates.

The intervention is in addition to usual rehabilitation. Usual rehabilitation includes, depending on need, half an hour per day of physiotherapy, occupational therapy and speech and language therapy. The intervention protocol is included as Additional file 1. At the end of the study, participants will be provided with details of community programs that people with stroke can attend after hospital discharge. When reporting study findings for publication, we will follow the Template for Intervention Description and Replication (TIDieR) checklist [37].

### Control group

Control participants will receive the same usual rehabilitation as the intervention group. Reflecting usual practice within the units, art made by previous participants will be available for viewing. After baseline assessment and randomization, the control group will receive from the study researcher a portfolio of work produced by previous participants of the Tayside CEI, with details of the community programs that people with stroke can attend after hospital discharge. Participants will be given the portfolio and invited to view it during their rehabilitation stay. Prior to outcome assessment, the study researcher will visit participants again to answer questions and to discuss options for community programs, if the person is interested.

### Trial procedures

Once participants meeting inclusion criteria have provided informed consent to participate, one of two study researchers will collect data indicating participant characteristics and will undertake baseline assessments. Stratification factors will be entered into the concealed online randomization system by the study researchers, who will inform the artists of group allocation. Participants in the intervention group will be invited to attend one individual and one group intervention session per week with the artist, for up to a maximum of eight sessions. The art portfolios will be delivered to participants allocated to the control group after baseline assessment.

A third researcher, blinded to group allocation and trained to administer outcome measures, will undertake outcome assessment with participants in the intervention group after eight art sessions, or at hospital discharge, if that occurs beforehand. The blinded researcher will undertake outcome measures with the control group at four weeks, or at discharge if that occurs prior to four weeks. Participants will be instructed not to reveal their group allocation to this researcher, but any episodes of un-blinding will be recorded. Three months after outcome assessment, the researcher will contact participants to arrange a follow-up assessment, which will be conducted in hospital or at the participants’ home, depending on discharge status. Nine participants from the intervention group and three from the control group, purposefully selected to reflect age, disability and gender, will also be invited to participate in audio-recorded in-depth interviews after follow-up assessment to evaluate their experiences of participation in the CEI, and the trial.

### Measures and measurement instruments

Preparatory qualitative work informed the trial design and enabled us to identify the types of outcome likely to be influenced by the CEI. Following on from the qualitative work, discussions with stroke survivors about their perceptions of undertaking the measures and how aphasia-friendly specific measures were, led to the final selection of measures. Outcome measures will be as follows:

#### Stroke-related health status

The Stroke Impact Scale [38] is a 59-item measure examining eight domains of health status: strength, hand function, ADL, mobility, communication, emotions, memory and thinking and participation and/or role function. Validity and reliability are established with Cronbach alpha coefficients of between 0.83 and 0.90 each of the eight domains [39–41]. Its’ test-retest reliability is good with intra-class correlation coefficients (ICC) of 0.7 to 0.92, excluding the domain of emotion, in which the correlation was lower (ICC = 0.57). High correlations were also demonstrated with the Mini-Mental State Exam, Barthel Index, Lawton Instrumental Activities of Daily Living, and the Motricity Index. Each item is rated on a five-point Likert scale indicating difficulty in completing the item. Summative scores for each domain range from 0 to 100. An additional question asks for a rating of 1 to 100 to indicate perceived degree of recovery from stroke. Scores can be added to provide a composite disability score.

#### Mood

The Positive and Negative Affect Scale [42] is a 20-item self-report measure comprising 10 positive and 10 negative affective descriptors. Measurement reliability has been examined; for the Positive Affect (PA) Scale, the Cronbach alpha coefficient was 0.86 to 0.90 and for the Negative Affect (NA) Scale it was 0.84 to 0.87 [39]. Over eight weeks the test-retest correlations were 0.47 to 0.68 for the PA and 0.39 to 0.71 for the NA. Strong correlation with measures of general distress and dysfunction, depression and state anxiety have also been reported [39]. Participants are asked to score the items on a five-point scale [1–5] ranging from ‘very slightly or not at all’ to ‘very much or extremely’ to indicate their emotions in the last week. The positive and negative affect scales each have a potential range of 10 to 50, where higher scores indicate higher affect. The scale has high validity and reliability for use in rehabilitation [43].

#### Self-esteem

The Visual Analogue Self-Esteem Scale [44] was designed to measure self-esteem following stroke. Cronbach alpha values between 0.74 and 0.84 have been reported, as have test-retest correlations of 0.80 and above [41]. The scale had concurrent validity with the Rosenberg Self-Esteem Scale (r = 0.74) when used in an aphasic stroke population [45]. The scale consists of 10 pairs of line drawings representing opposing poles of self-descriptive constructs. Written labels above the drawings explain the constructs. Participants are asked to rate the extent to which the constructs reflect perceptions of themselves on a scale ranging from one to five. The item responses are summed to provide a total score ranging from 10 to 50.

#### Recovery locus of control

The Recovery Locus of Control Scale is a nine-item scale designed to measure perceptions of control over recovery [46]. Scale items measure internal and external beliefs. Items are scored on a five-point Likert scale, with no control represented as one and complete control as five. The scores of all items are added to give a measure of the strength of internal control between nine and 45, with 45 indicating maximum internal control. Validity and internal consistency with stroke survivors has been established [46]

#### Hope

The Trait Hope Scale [47] is a 12-item measure that comprises two subscales: a four-item agency subscale (for example, ‘I energetically pursue my goals’) and the four-item pathways subscale (for example, ‘There are lots of ways around any problem’). An additional four items are distracters. Both subscales have good internal reliability, with Cronbach’s alphas ranging from 0.70 to 0.84 for the agency subscale, and from 0.63 to 0.86 for the pathways subscale [47, 48], and has concurrent validity when compared to instruments examining similar psychological constructs, such as optimism and self-efficacy. Items are scored on a four-point Likert scale, using anchors ranging from ‘1 = definitely false’ to ‘4 = definitely true’.

#### General self-efficacy

The General Self-Efficacy Scale [49] is a 10-item scale that is designed to assess optimistic self-beliefs to cope with a variety of difficult demands in life. Cronbach’s alpha values range from 0.76 to 0.90, with the majority of values in the high 0.80s, illustrating good criterion validity. Responses are made on a four-point scale scoring between one and four. Responses are summed to give a total score out of 40, indicating maximum self-efficacy. The scale has been widely used with stroke populations [50, 51].

#### Self-efficacy for art

To assess self-efficacy and perceived difficulty for art expression we will ask two single item questions, generated following an established procedure for examination of self-efficacy [52]. The questions are: 1. How confident are you that you can express yourself through art activities? And 2. How difficult do you find it to express yourself through art activities? Self-efficacy for art expression will be scored on a seven-point vertical visual analogue scale with one as least confident/difficult and seven as most confident/difficult.

### Outcome measures

The proposed outcome measures were selected to reflect clinical and stroke-related outcomes likely to be affected by the intervention. The final selection of primary outcome for a definitive trial will be determined by the findings of this feasibility trial, but our exploratory work indicates that stroke-related health status measured by the Stroke Impact Scale, is likely to the primary outcome measure. The scale examines communication, participation in life roles, physical status and mood, which were key outcomes indicated by the qualitative work as important benefits of art participation. In addition, the Positive and Negative Affect Scale was selected to investigate positive emotions such as enthusiasm and alertness, which our qualitative work and other recent studies [26] indicate might be influenced by art participation. Similarly, the qualitative work demonstrated that stroke survivors perceive improved self-esteem through art participation, but more specifically from the final stage of the intervention, in which the artwork is displayed and viewed by others. We therefore included the Visual Analogue Self-Esteem Scale [45] as an outcome measure.

To examine underlying psychological constructs that appear to explain how art might influence improvements in the above outcomes, we have also included other secondary outcome measures. Participants in the qualitative study indicated these as important. Survivors suggested that recovery of upper limb function and communication occurred because art provided them with the opportunities to develop their own control of these aspects of stroke recovery in ways that other rehabilitation did not. The art itself provided ways in which survivors could use their upper limbs and try out activities for themselves. Interactions with the artists and within the group provided an environment in which survivors could practice and regain control over their communication. We have therefore included as an outcome the recovery locus of control [46], which indicates internal or external locus of control over recovery and will provide a measure of change in control as a result of art participation.

Confidence that personal rehabilitation goals and art-specific goals could be achieved was generated through the opportunities for skill acquisition that art provided. Confidence was inferred by the qualitative study as a route by which art participation improved recovery, self-esteem and mood. We therefore included a measure of general self-efficacy [49–52] and one that examines self-efficacy for art [46], to evaluate whether art improves self-efficacy and influences more distal outcomes, such as stroke recovery. Perceptions of difficulty can also influence self-efficacy therefore a single-item measure to assess difficulty has been included, as suggested by Francis *et al*. [52].

Hope appears to predict better rehabilitation outcomes. Hope occurs when a person perceives routes to achieving goals and has motivation to access those routes [53, 47]. It has been hypothesized that in rehabilitation hope buffers negative emotions because the individual believes that goals can be achieved despite obstacles [48, 54]. Hope was another concept that our qualitative interviews suggested was developed through art participation. We therefore included the Hope Trait Scale [47] as a secondary outcome. The scale measures hope agency thinking, which represents a patient’s wilful sense of determination to meet goals, and pathway, which reflects a patient’s perceived availability of ways to attain a goal.

This is a pilot trial, therefore as well as clinical and psychological outcomes, we are also collecting data on other outcomes that relate to the feasibility of conducting a definitive trial. We will collect data and evaluate trial recruitment rates, the proportion of participants who prefer to be randomized to art and retention and dropout rates. We will examine the variability of scores on outcome measures and completion rates and ease of use of the chosen measuring instruments at each assessment point. We will also undertake comparison between the measures to determine which are more appropriate for use in a definitive trial. Finally, we will evaluate with participants and staff, the acceptability and feasibility of undertaking the trial in rehabilitation settings.

### Sample size calculation

This is a feasibility trial therefore we have not undertaken a formal sample size calculation. A sample size of 40 participants in each group will be adequate to provide estimates of direction and magnitude of any effects, and to provide an estimation of variability for later sample size calculation. Previous literature shows that a sample size of between 30 and 36 per arm is adequate for a feasibility trial [55]. We based our sample size on that information, on our knowledge of likely participants to the art program, and on information from our previous experience of likely drop-out rates from stroke rehabilitation trials.

### Fidelity

The project processes will be facilitated by the use of clear written information: guidance to rehabilitation staff on the stroke units about identifying eligible patients, clear protocols for the artists, use of individual patient case reports to log each stage of the project process and training for all researchers in outcome measures. The project researchers will be in regular contact with the stroke units to provide support and to deal with any problems.

### Withdrawal from study

There will be five points at which a participant may withdraw from the study - prior to completing baseline questionnaires, prior to commencement of the CEI, prior to completion of the CEI, after completion of the CEI but prior to completion of outcome measures and prior to completing the three-month follow-up assessment.

We do not anticipate any harms occurring as a result of participation in this study, however, we remain alert to that possibility. Withdrawal will occur if the participant requests it, if the participant is unable to participate in any aspect of the study or if there is any indication that participation is causing harm of any kind.

### Data confidentiality and management

All records will be securely stored in locked cabinets in areas with restricted access. All administrative and data records will be identified by a coded identification number to maintain participant confidentiality, and these will be kept separately from records containing names and other personal identifiers. All databases will be secured with password-protected access systems. TCTU will be responsible for data management, data quality assurance, backup, business recovery and statistical analysis. A formal data monitoring committee was not required because this is a feasibility trial.

### Statistical analysis

The Montreal Cognitive Assessment and the National Institutes of Health Stroke Scale will provide screening data and indicate baseline status. Other baseline characteristics to be collected are: age, sex, handedness, ischemic or hemorrhagic stroke, side of hemiplegia, Barthel Index, Aphasia Severity Rating Scale [56] and use or not of psychotropic medication. Preference for randomization to the intervention group (participating in art) or the control group (viewing art) will be assessed after randomization.

Data analysis will be undertaken blind to group allocation. The primary analysis will be based on the intention-to-treat principle. Proportions of missing data will be examined to inform decision-making about selection of outcome measures for the definitive trial. Transformations of the outcome variables will be used where necessary if these are not normally distributed. All analyses will incorporate the stratification factors.

Data will indicate, with confidence intervals, the proportion of patients choosing or refusing participation in the CEI and will allow calculation of recruitment rates for a future definitive trial. Data about the proportion of study dropouts will also provide information that will inform a definitive trial. Summary data from measures will indicate potential variability, magnitude and direction of difference between groups at the end of intervention and at three-month follow-up.

To examine the effects of the intervention, a repeated measures analysis using mixed models will be conducted for outcomes, although these will not involve definitive hypotheses testing. Adjustment for baseline factors and mediating variables will be undertaken by adding these as covariates to give an idea of which factors are important in determining outcome. This will indicate where the intervention effects are likely to occur and will generate power calculations for a future definitive RCT. The measure with greatest difference as well as most relevance in the feasibility study will be selected as primary outcome measure, whilst taking into account proportions of missing data. Missing data will be examined for randomness, and if appropriate, multiple imputations will be conducted to replace missing values. Intention-to-treat analysis based on outcome evaluation of cases as randomized will be conducted to examine any important differences in findings. Reporting of results will adhere to Consolidated Standards of Reporting Trials criteria for the reporting of randomized controlled trials [57].

### Qualitative phase

To ascertain participants’ views and experiences of the remodeled CEI, 12 participants from both stroke units will be purposefully selected for in-depth interview. The sample will include three participants who were randomized to the control group. It is likely that experiences of the CEI will be influenced by participants’ gender, age, disability and the artist involved in intervention delivery. Therefore, we will purposively select male and female participants of different age groups and with different levels of disability. The interviews will explore experiences, beliefs and attitudes about the CEI, the values and meaning placed on engagement in the CEI and its perceived effects. The interviews will also seek to examine how participation in the CEI is translated into meaningful benefits. Finally, the ways in which the CEI was perceived in relation to other forms of rehabilitation will be explored along with the experience and acceptability of participation in a trial.

The consent process at study recruitment will invite participants to agree to be contacted at the end of the study for participation in an interview. Participant interviews will be carried out after the three-month follow-up assessment. Our pre-study qualitative work showed that a total of approximately 12 participants is sufficient to reach the point at which no new findings emerge [58]. Focus groups with staff on each unit and interviews with artists will be conducted after the intervention phase of the study is complete. Written informed consent will be obtained at that time. One focus group of approximately six staff who have been involved in supporting the trial will be conducted at each site and will include staff from a range of disciplines, including support staff.

With participants in the intervention group we will explore their reasons for and experiences of taking part in the trial and their perceptions of acceptability and perceived usefulness of the CEI. We will examine the perceived value and meanings they ascribe to participation in the CEI and the impact of their preference for art on trial participation and perceptions of the CEI. Finally, we will ask about their perceptions of art participation as part of rehabilitation and any impact the CEI participation has had on their life after rehabilitation.

With the control group we will explore their reasons for and experiences of taking part in the trial and the impact that preference for art had on their participation. We will also examine their perceptions of art as part of rehabilitation and any changes that they made to their behavior as a result of trial participation. Finally we will explore any impact that the control intervention had on their life after rehabilitation.

We will ask staff and artists about their experiences and their perceptions of acceptability of all elements of the trial. We will also explore any perceived impact the trial had on usual practice and examine how they view the role of the CEI as part of rehabilitation. Finally, we will examine their views about the perceived effects the intervention had on participants.

### Data management and analysis

To ensure anonymity of data, interview transcripts will be given a study code and identifiable data within transcripts will be changed to ensure anonymity. Nvivo 9 (QSR International, Daresbury, United Kingdom) will be used to manage the data. We will use the Framework Approach, which was developed by the National Centre for Social Research in the UK, and is widely used for analysis of policy and health related qualitative data [59]. Framework provides a systematic approach to classification and organization of data in terms of emerging themes and patterns. Analysis will be iterative. New issues identified from interviews will be iteratively included in subsequent interviews for further exploration. A coding frame will be developed from data within the transcripts and from key psychological concepts identified from the pre-study interviews. We will apply the coding framework to all transcripts and emerging categories and themes will be added to the framework as they emerge. After all transcripts have been coded, the coding frame will be refined by review of each category, and by seeking association between themes and grouping them under more comprehensive, higher order themes.

To ensure trustworthiness of the analytical process, two researchers will check data coding for accuracy and we will examine our interpretations by constantly searching for alternative explanations. We will also discuss emerging themes and patterns and concepts with the wider research team. We will use findings from the qualitative research alongside findings from the feasibility trial to design the full RCT.

### Study timeline

The total study duration is 24 months. The study start date was the 1 March 2013. The pre-trial qualitative work and definition of trial outcome measures was conducted between 1 March 2013 and 31 July 2013. Trial recruitment is between 29 July 2013 and 30 July 2014. Baseline data is being collected from 9 July 2013 until all patients are recruited. Intervention delivery is between 29 July 2013 and 31 July 2014. End of intervention outcome assessment is between 22 August 2013 and 31 July 2014, with follow-up outcome assessment between November 2013 and November 2014. Qualitative interviews of participants who have completed the intervention are between January 2014 and November 2014. Trial data analysis will start in November 2014 and the planned study end date is the 28th February 2015.

### Dissemination plans

Two former stroke patient participants are members of our expert steering group as equal contributors and will advise on dissemination strategy. As per ethics committee requirements, we will write to all participants informing them of the study findings. To disseminate information to practitioners, we will also present our findings to acute stroke units, appropriate rehabilitation centers and relevant arts in health organizations across Scotland. We will also present findings through lectures at therapy meetings and local and national special interest groups, such as the Stroke Association, Chest, Heart and Stroke Scotland and others. To disseminate findings to academic audiences, we plan to publish our findings in high-impact peer-reviewed medical, therapy, and arts in health journals. We will also present findings at national and international therapy, medical and arts in health conferences and report findings in the clinicaltrials.gov database.

## Discussion

There is growing interest in the role that participation in the arts can provide in healthcare settings. Evidence is also emerging that art participation can augment rehabilitation, help to address the psychosocial effects of stroke and prepare survivors for fulfilling lives after rehabilitation. Providing such an intervention in rehabilitation settings is potentially costly, therefore its effects should be evaluated in the same way as other healthcare interventions, using robust scientific methods. Furthermore, assuming that art participation is a valuable adjunct to rehabilitation, it is vital that we seek to understand how it works and the outcomes that it influences. In line with other rehabilitation interventions, such understanding will enable us to refine the intervention to target it at those most likely to derive its known benefits.

However, the creative and apparently subjective nature of art participation means that designing a trial to test its effects is challenging. Our preparatory qualitative work based on the Medical Research Council’s (MRC) Framework for complex intervention design has enabled us to define the stages of the CEI and to provide a framework that is replicable, based on the core elements of an existing program. Our study endorses the importance of qualitative preparatory work as a precursor to trial design for complex interventions. This work enabled us to define the ways in which the intervention appears to work and the outcomes it appears to influence. The feasibility trial will enable us to undertake preliminary testing of these hypotheses and to determine feasibility issues associated with undertaking a trial of this nature in rehabilitation settings. Furthermore, qualitative interviews with participants at the end of the trial will enable us to confirm or disconfirm the effects of the CEI and our selection of outcome measures, and to refine the design of a full-scale definitive RCT.

To our knowledge, this is the first study aimed at examining the feasibility of using trial methods to evaluate the effectiveness of an art intervention for stroke survivors in rehabilitation. We hypothesize that participation in a CEI will improve the psychosocial outcomes for stroke survivors during and after rehabilitation. If shown to provide effect sizes, indicative of meaningful effects this intervention has the potential to address aspects of stroke recovery previously not addressed in rehabilitation, and will provide convincing evidence of benefits to healthcare providers and funders.

### Trial management

A trial management group has been convened with the support from the United Kingdom Clinical Research Network registered Tayside Clinical Trials Unit. The group will meet regularly throughout the trial period to make decisions and to support progress. The management group involves Dr. Jacqui Morris (study Principal Investigator), Chris Kelly (Trial Manager) and co-applicants Professor Brian Williams, Professor Thilo Kroll, Professor Peter Donnan, Professor Gillian Mead and Dr. Sara Joice. Dr. Madalina Toma (study research associate) and Drs Fiona Hogarth and Petra Rauchaus from TCTU also sit on the trial management group. The management group has agreed that we will operate within the framework suggested in the MRC Guidelines for good clinical practice in clinical trials [49].

## Trial status

Recruitment started in July 2014 and finished in August 2014.

## Authors’ information

JM is a physiotherapist and senior researcher with a background in stroke rehabilitation and recovery research. CK is an artist and the manager of Tayside Healthcare Arts Trust. He conceived the intervention and with JM, developed the study design. BW is a social scientist with an extensive portfolio in qualitative research in public health and primary care who has conducted a number of clinical projects and has experience in general practice behaviour and guideline interpretation and implementation. PTD is a bio-statistician with experience in clinical research and RCT techniques and is co-director of TCTU. GM is a geriatrician with extensive experience and an extensive portfolio of research and clinical trials in stroke recovery and physical activity. TK is a psychologist with a portfolio of research focused on disability, rehabilitation and public health and has a particular interest in community engagement. SJ is a health psychologist and has a research background examining health behaviors associated with life after stroke. MT is a qualitative researcher and is employed as a research associate on the grant.

## Electronic supplementary material

Additional file 1: **Creative engagement intervention: artists’ protocol.** (DOC 202 KB)

## Abbreviations

CEI: Creative engagement intervention
RCT: Randomized controlled trial
TCEI: Tayside creative engagement intervention
TCTU: Tayside Clinical Trials Centre
MOCA: The Montreal Cognitive Assessment
NIHSS: The National Institutes of Health Stroke Scale
PA: Positive Affect
NA: Negative Affect, NRC, Medical Research Council.
