# Supplementary material for: Feasibility study of the effects of art as a creative engagement intervention during stroke rehabilitation on improvement of psychosocial outcomes: study protocol for a single blind randomized controlled trial: the ACES study
Source: Trials. 2014 Sep 28;15:380. doi: 10.1186/1745-6215-15-380 (PMC4190489; doi:10.1186/1745-6215-15-380)
Supplement: Supplementary file 1 — Additional file 1: Creative engagement intervention: artists’ protocol. (DOC 202 KB) [file 13063_2014_2248_MOESM1_ESM.doc]

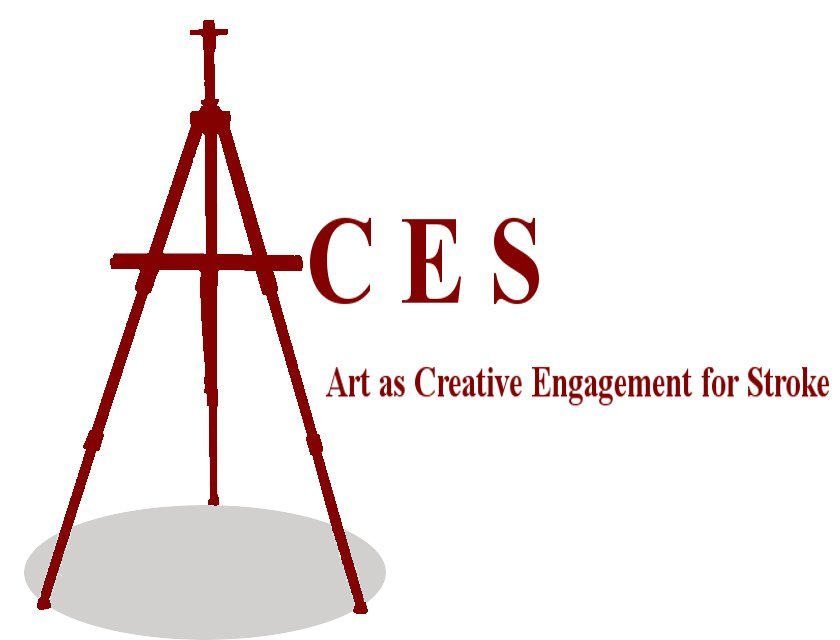


**INCLUDEPICTURE "http://t0.gstatic.com/images?q=tbn:ntRspU7mSBn1jM:http://www.dundee.ac.uk/pamis/photos/UoD4_process_logo.jpg" \* MERGEFORMATINET**


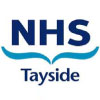

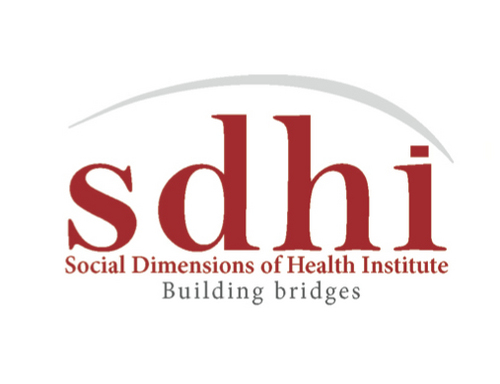

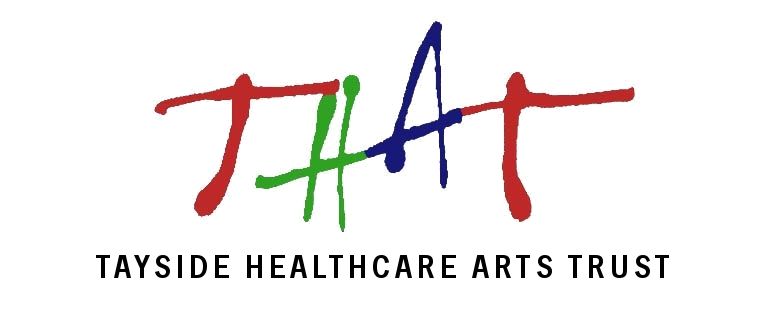

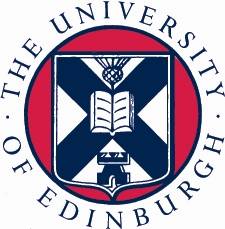

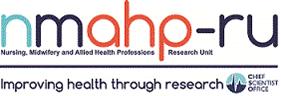


**Creative Engagement Intervention: Artists’ Protocol**

The Artists’ Protocol is designed to ensure that the artists delivering the Creative Engagement Intervention (CEI) as the key element of the ACES Study are both confident in, and comfortable with, the definition, explanation and guidance for their role.

Pilot data from THAT, alongside the qualitative analysis from Phase 1 of the study has informed the establishment of the following terms of the protocol.

The artists will provide two different sessional engagements to participants. These will be 1:1 sessions approximately 1hr in duration and group sessions approximately 1.5 hr in duration. Phase 1 analysis has shown that these are considered optimum engagement times for both these types of sessions.

Up to 5 x 1:1 sessions will be delivered to study participants during one mid-week day (as agreed with the Artist and their allocated Unit) and 1 x group session (with a maximum of 5 participants) will be held on Saturday mornings.

The artists will work with a participant for up to a maximum of 8 sessions. It is anticipated that the balance of these sessions would normally be 4 x 1:1 sessions and 4 x group sessions, however this will be at the discretion of the Artists, based on their assessment and judgement of the most appropriate engagement process for each individual participant. Session attendance will be recorded by the Artist as will progress and movement across the CEI component stages. Appropriate paperwork for recording this information will be provided. The Artist will photographically record all significant creative outputs made.

The Artist will respect protected mealtimes and other health management priorities identified relevant to either a particular participant or the management of the unit.

The Artist will be treated as a member of the multidisciplinary team, be provided with equal access to participants and supported in achieving the goals of the CEI research.

The Creative Engagement Intervention has been identified as having five component stages (see table 1, below), which have been refined though the Phase 1 analysis. These components map the participant’s likely journey with the Artist

**Table 1.CEI Component Stages**

| 1. Meeting with Artist, discuss interests, stroke and define initial creative goals. |
| --- |
| 2. Introduction to materials and mark making (drawing/collage/printing/painting/mixed media  techniques). |
| 3. From materials and mark making to developing personal project ideas. |
| 4. Developing personal project ideas into creative finished pieces. |
| 5. Review of completed work, mounting and display of work, future plans. |

Although the five stages of the CEI indicate the pathway for the participant, it does not prescribe a fixed linear experience. It is recognised that participants will progress at different speeds and in different ways. Components may be repeated or retuned to or may be addressed simultaneously (stages 1 & 2). Participants may progress rapidly and move from stage 5 back to stages 2, 3 or 4 to progress new work or experience new materials or processes.

CEI Component Stages:

Stage 1 Meeting with Artist, discuss interests, stroke and define initial creative goals.

This is a vital first step in building a therapeutic relationship with the participant and will be best achieved through a 1:1 session. It is important to engage participants on their terms of interest whilst also gaining direct knowledge about their state of health and impairments as result of their stroke. This should then be moved to discuss expectations and artistic goals and possible first creative steps. The participant profile should be completed at this stage.

Stage 2 Introduction to materials and mark making (drawing/collage/printing/painting/mixed-media techniques).

Depending on the interests and initial goals of the participant, the Artist will select appropriate materials, tailored to participants’ impairments, and begin a playful exploration of ability. This is essential to ascertain the extent of deficit reduction in functional ability and to judge the accuracy of participants’ expectations. It is also the first opportunity to show that the handling of creative materials can be a sensory and satisfying experience.

Stage 3 From materials and mark making to developing personal project ideas.

Having engaged them through the handling of the materials the artist will then guide the participant on how to use the materials to consider content or subjects that are of personal interest to them. This may reveal greater personal detail about the participant and if appropriate to share may strengthen the social aspect of group sessions. This may require some simple research on the part of the artist but equally may involve the participant or their family in providing particular reference material. This places the participant in the position of expert and director which can empower them to take greater control of their output.

Stage 4 Developing personal project ideas into creative finished pieces.

The Artist will guide and assist the participants to an appropriate creative conclusion for individual pieces of work. The participant will control and direct the expression of content and the artist will instruct and facilitate the creative process and interpretation to allow that to happen. Ideas may have more than possible means of expression. Participants may wish to make more than one attempt at rendering their chosen expression. Creative work generally leads to more creative ideas.

Stage 5 Review of completed work, mounting and display of work, future plans.

The achievement of one or more completed creative works provides the tangible expression of the creative experience for the participant. The Artist will discuss the completed work with the participant, reviewing the process of making and the expression of content. This should then lead to discussion of further possible ideas which can be progressed with the artist (stage 2,3 or 4) or whether they will have any interest in pursuing creative opportunities after discharge. The Artist will ensure that all completed works are mounted appropriately and if agreed by the participant displayed within the ‘Gallery Wall’ facility within the unit. This shows respect and regard for the participant’s work, which promotes confidence and self esteem. The public sharing of the work can also act as a social and communication stimulus and alter perceptions of ability and identity.
